# Establishment and comparison of three sublines from a human uterine carcinosarcoma cell line, ESCA

**DOI:** 10.1007/s13577-025-01225-8

**Published:** 2025-06-02

**Authors:** Yixiu Long, Xuan Pei, Hongyu Liu, Xueyan Ouyang, Wei Jiang, Huijuan Yang

**Affiliations:** 1 Department of Gynecological Oncology, Fudan University Shanghai Cancer Center, Fudan University, Shanghai, 200032 China; 2 Department of Oncology, Shanghai Medical College, Fudan University, Shanghai, 200032 China

**Keywords:** Uterine carcinosarcoma, Cell line, ESCA, TP53, TRRAP

## Abstract

The pathogenesis of uterine carcinosarcoma (UCS) remains unclear due to a few mature cell lines. Herein, we established a new cell line, ESCA, from a Chinese woman. Especially, three sublines, named ESCA-2, ESCA-3, ESCA-5, were isolated based on the rate of cells’ different sedimentation. All ESCA cells have been subcultured for more than 60 generations. ESCA sublines display different cell morphology and growth characteristics, as well as have different sensitivity to chemotherapeutic drugs. ESCA was most sensitive to paclitaxel and carboplatin, while ESCA-2 was most sensitive to ifosfamide. Besides, ESCA showed severe chromosome karyotype abnormalities and abnormal number of chromosomes. Whole exome sequence showed ESCA and its sublines, as well as tissue block shared similar single nucleotide variants, such as *TP53*, *TRRAP* mutations, while relatively large differences in mutational signature abundance. When all ESCA cells were xenotransplanted subcutaneously into BALB/c-nu mice, they developed into tumors that resembled the original tumor with positive AE1/3 and Vimentin in immunohistochemical staining. Interestingly, the transplanted tumor from ESCA-5 proliferated fastest with a relatively low level of glucose uptake evaluated by micro-PET/CT scanning. Taken together, ESCA and its sublines may be valuable tools to explore the molecular mechanism of UCS**.**

## Introduction

Uterine carcinosarcoma (UCS) is a highly aggressive biphasic malignancy, mixed with carcinomatous and sarcomatous elements. Although UCS accounts for only 6% of all gynecologic cancers, it contributed to about 16% of deaths caused by uterine malignancies [1, 2]. For patients with early-stage UCS, abdominal hysterectomy is the best choice of treatment and the 5-year overall survival (OS) is 58%. However, approximately one-third of UCS patients are diagnosed at an advanced stage, primarily managed with chemotherapy, resulting in a dismal 5-year OS rate ranging from 9 to 22% [3–5]. Despite targeting the known dysfunction pathways, such as *ERBB2*, is rapidly evolving and generating hope for the treatment of UCS, prognosis of patients with UCS has limited improvement. Consequently, it is urgent to explore novel treatment regimens [6].

Histologically, UCS is characterized by the presence of high-grade malignant epithelial and mesenchymal components. The epithelial elements closely resemble established histologic sublines of endometrial cancer, such as endometrial or serous carcinoma, while the mesenchymal elements may exhibit cellular characteristics homologous or heterologous to those of the uterus [7, 8]. Homologous components often resemble high-grade undifferentiated sarcoma or fibrosarcoma of the uterus, whereas heterologous components typically resemble rhabdomyosarcomas or chondrosarcomas [9]. Historically, UCS was classified as a sarcoma, but recent studies have suggested that it shares more characteristics with endometrial carcinoma (EC). UCS shares the patterns of chromosomal instability and mutational spectra with EC [10, 11]. Levine et al. demonstrated that UCS had extensive copy number alterations and highly recurrent somatic mutations, such as *TP53* (91%), *PTEN* (19%), *PIK3 CA* (35%), *PPP2R1 A* (28%), *FBXW7* (28%) and *KRAS* (12%), by a comprehensive analysis of UCS from molecular insights, which bears similarity to that of EC [10, 12]. Previous studies analyzed micro-dissected epithelial and mesenchymal components from some individual patients of UCS, and suggested that they presented common chromosomal alterations and mutations, implicating a monoclonal origin [13]. From above perspectives, it is difficult to explain why the prognosis of UCS is poorer than that of EC. Therefore, the establishment of a mature UCS cell line is crucial for understanding the molecular basis and exploring novel treatment strategies for this malignancy. However, there are very few cell lines of UCS, limiting research progress in UCS. Herein, we report the establishment of a novel UCS cell line, named ESCA, encompassing the isolation and characterization of three distinct sublines.

## Methods

### Acquisition of specimens

A 58-year-old Chinese woman consulted the department of Gynecological Oncology, Fudan University Shanghai Cancer Center with a chief complaint of persistent post-menopausal vaginal bleeding for 5 months. PET/CT examination suggested that uterine malignancy tumor, with no evidence of metastasis. Subsequently, she underwent surgery (including subtotal hysterectomy, bilateral adnexectomy, omentectomy, as well as pelvic and para-aortic lymphadenectomy) and the tumor localized to the uterine cavity, with no gross or microscopic involvement of bilateral ovaries, fallopian tube or lymph node. The original tumor exhibited a biphasic morphology in hematoxylin–eosin (HE) staining: glandular structures are irregularly branched and crowded, resembling endometrioid adenocarcinoma and the stroma was dominated by spindle-shaped cells with marked nuclear atypia. Immunohistochemical analysis revealed that tumor tissues were positive for Vimentin, Desmin, ER, CD10 and negative for p53, MyoD1, WT1. Taken together, the patient’s tumor comprised endometrioid adenocarcinoma-like and homologous sarcomatous components. She received 6 cycles of adjuvant paclitaxel (175 mg/m^2^) plus carboplatin (AUC = 5) chemotherapy to reduce the risk of recurrence and metastasis. The adjuvant treatments were finished on 7 September 2018, and follow-ups were conducted regularly. Unfortunately, the chest CT showed multiple nodules on the right lung after 5 months, which was confirmed that the pulmonary nodules were UCS metastatic by a CT-guided needle biopsy of the lungs. The patient refused chemotherapy for financial reasons. Shortly thereafter, she died for rapid tumor progression. Informed consent was obtained from the patient before surgery according to institutional guideline (FUSCC050432–4-1212B).

### Establishment of cell line

Fresh tumor tissue (about 1 cm^3^), extracted by surgery, was rinsed several times with phosphate buffer solution (PBS) to get rid of connective and necrotic tissues. The sample was then finely minced and dissociated in 0.02% EDTA/PBS (–) and 0.1% trypsin (BD, MD, USA) for 30 min at 37 °C. The digested solution was filtered using a 200-mesh sieve. The filtrate was allowed to stand for 30 min, followed by collection of the supernatant into another clean centrifuge tube. The remaining precipitate was cultured in complete growth medium (RPMI 1640 medium with 15% fetal bovine serum, 100 IU/mL penicillin, 50 µg/mL streptomycin, 1% sodium pyruvate, 1% HEPES buffer and 1% nonessential amino acids) and incubated at 37 °C with 5% CO_2_ and marked as ESCA-1. The remaining supernatants continued to repeat the above procedure, a total of 4 times, respectively, recorded as ESCA-2, ESCA-3, ESCA-4 and ESCA-5. Therefore, we got five cell sublines based on the rate of cells’ different sedimentation rates. Unfortunately, ESCA-1 subline containing few tumor cells was failed to grow in primary culture. The remaining four sublines cell lines were purified in the process of passage based on the method of enzyme digestion and differential adhesion. Regrettably, the subline of ESCA-4 gradually decreased and completely disappeared during the purification process. However, the other three sublines remained stable throughout the passages. Upon reaching the 10 th passage, the three sublines were combined and named ESCA. Finally, a new cell line of ESCA and three sublines were established successfully with more than 60 generations. In addition, ESCA cells were sent to China General Microbio-logical Culture Collection Center for preservation (No. 17281) in January 2019.

### Growth characteristics

ESCA and three sublines were seeded into 6-well plates at a density of 1 × 10^5^ cells in 2 mL of culture medium and incubated for 10 days. Viable cells were counted in triplicate every 2 days. And the culture medium in remaining plates was changed every 3 days. After five consecutive measurements, the cell proliferation curve was obtained and the cell doubling time was calculated.

### Chromosome analysis

Chromosome analysis was performed in ESCA cells, as described in a previous study. Treated with 0.05 μg/mL colchicine for 2 h at 37 °C, the cells were digested, resuspended and placed in a hypotonic solution (0.075 M KCl) for 30 min at 37 °C. Then fixed with a methanol–acetic acid (3:1) solution and kept overnight at 4 °C, the cells were digested with 0.1% trypsin solution for 15 s at room temperature. Finally, stained with 3% Giemsa, the specimens were loaded into scanner for G-band karyotyping. The histograms of the chromosomal distribution were determined based on more than 50 metaphases.

### Short tandem repeat (STR) detection

The STRs of ESCA and its sublines were analyzed to authenticate the established cell lines. Then the STR profiles were compared with those recorded in the American National Standard Institute (ANSI/ATCC ASN-0002-2011 Authentication of Human Cell Lines: Standardization of STR Profiling).

### Transwell and wound healing assays

All ESCA cells (2 × 10^5^) in 200 µL RPMI-1640 without fetal bovine serum were, respectively, placed in the upper chamber of Transwell inserts (24 wells, 8 µM pore size; Corning Costar), which were coated with Matrigel (Corning, 356,237). And the lower chambers were loaded with 500 µL RPMI-1640 with 15% fetal bovine serum. Incubated at 37 ℃ for 48 h, cells were removed from the upper chamber and cells on the lower membrane surface were fixed with 4% formalin, stained with 0.1% crystal violet for visualization, and counted under a microscope (Leica). For the wound healing assay, ESCA and its subline cells were, respectively, seeded 6-well plates (3 × 10^6^ cells/well). Then, a line was scratched across the center of the well after adhesion of the cells to the well. After 24 h, the cells were washed three times with PBS and quantified using a microscope.

### Immunofluorescence and immunohistochemistry

ESCA and its sublines cells were seed on to sterile glass slides, incubated 48 h, washed 3 times with PBS, fixed with 4% paraformaldehyde for 15 min, air-dried, and treated with 0.5% Triton X-100 for 20 min. The slides were then incubated with the following primary antibodies overnight at 4 °C: Pan-Keratin (AE1/3) Mouse mAb (1:100; Cell Signaling Technology, Inc) and Vimentin Rabbit mAb (1:100; Cell Signaling Technology, Inc). Subsequently incubated with the following secondary antibodies for 30 min in the dark at room temperature: Cyanine 3-conjugated goat anti-mouse immunoglobulin G (IgG) or Alexa Fluor 488-conjugated goat anti-rabbit IgG (both diluted at 1:500, Servicebio). Nuclei were stained with DAPI (Beyotime). Images were obtained under a confocal laser scanning microscope (FV3000 confocal microscope).

For immunohistochemistry, the slices, after embedding in paraffin and sectioning, were covered with the following antibodies: Pan-Keratin (AE1/3) Mouse mAb (1:100; Cell Signaling Technology, Inc.) and Vimentin Rabbit mAb (1:100; Cell Signaling Technology, Inc.). Finally, the microscopic slides were reviewed by a senior gynecology-dedicated pathologist (Prof. Yang).

### Anticancer drug susceptibility tests of in vitro

ESCA and three subline cells (around 5 × 10^3^ cells) were inoculated into 96-well plates at 37 °C for 24 h. The next day, the medium with anticancer agents at different concentrations replaced the used culture medium. The concentrations of each drug were as follows: 0.0001–500 μM paclitaxel (Med Chem Express Industries, China.), 0.01–1600 μM carboplatin (Med Chem Express Industries, China) and 0.001–10 mM ifosfamide (Med Chem Express Industries, China.). After 48 h, the medium with drugs were replaced by medium containing CCK-8 (10 μL) and incubated for 2 h. Then, absorbances of cells were measured at a wavelength of 450 nm (OD 450) by a microplate reader (Synergy H4, Bio-Tek) to obtain a dose–response curve for each agent.

### Whole-exome sequencing (WES) of ESCA cell and patient’s tumor tissue

Whole-exome sequencing (WES) was performed on all ESCA sublines and patient using DNA from tumor sample and cells. The operation was performed according to Illumina’s standard procedure (effective tumor depth of 300×, blood sample of 100×). First, DNA was extracted using the QIAamp DNA FFPE Tissue Kit Print (Qiagen, Hilden, Germany) according to the manufacturer’s protocol. A paired-end DNA library was prepared according to the manufacturer’s instructions (Agilent, Santa Clara, CA, USA). Samples with a total amount greater than 0.6 µg were used for library preparation. Genomic DNA was fragmented by Covaris sonication to a length of 200–300 bps. The ends of DNA fragments were repaired, and Illumina Adaptor was added (Fast Library Prep Kit, iGeneTech, Beijing, China). The DNA fragments were end-polished, A-tailed and ligated with the full-length adapter. After the sequencing library was constructed, whole exomes were captured with the AIExomeV2 (T192 V1 T) Enrichment Kit (iGeneTech, Beijing, China) and sequenced on an Illumina platform (Illumina, San Diego, CA, USA) with 150 bp paired end reads. Raw reads were filtered to remove low-quality reads using Fast QC. Clean reads were mapped to the reference genome GRCh37 using BWA. After removing duplications, single nucleotide variants (SNVs) and insertions and deletions (indels) were called and annotated using the Genome Analysis Toolkit (GATK) based on dbSNP build 150. All variants were annotated with ANNOVAR [14].

### Heterotransplantation

All the procedures were approved by the laboratory animal center, cancer hospital affiliated to Fudan University Shanghai Cancer Center (FUSCC-IACUC-2023369). 30 BALB/c-nu mice (4–6 weeks, Shanghai SLAC Laboratory Animal, Shanghai, China) were fed under SPF conditions. After adaption of 1 week, mice were divided into four groups and injected subcutaneously into the dorsal flanks with four kinds of ESCA cells (1 × 10^7^ cells, each mouse), respectively. Tumor growth was measured every 5 days and calculated by longest diameter × shortest diameter^2^ × 1/2. Magnetic resonance imaging (MRI) of transplanted was performed 22 days after the tumor was visible to the naked eye. Micro-PET/CT scanning was performed 25 days to evaluated tumor metabolism. Then, the mice were raised until the 45 days and euthanized. Finally, the tumors were harvested for analysis.

### Statistical analysis

All experiments, except the animal experiments, were repeated three times. Differences among groups were analyzed using one-way ANOVA with Tukey’s post hoc test for normally distributed data. GraphPad Prism 8 software (San Diego, CA) was used for the statistical analysis. The statistical significance is defined as *P* < 0.05.

## Results

### ESCA sublines display different cell morphology and proliferation rate

**Fig. 1:**
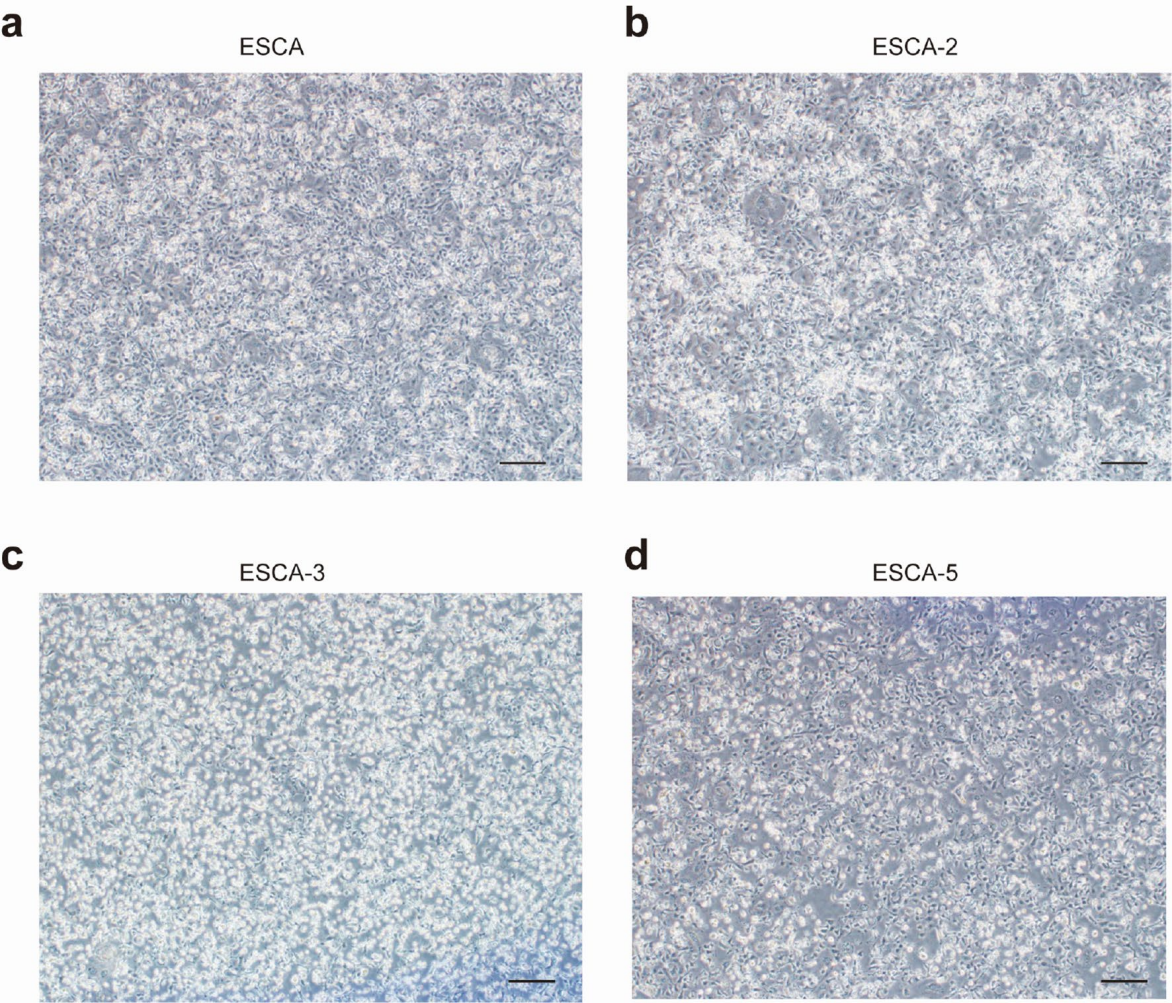
Cell morphology of ESCA and its sublines in phase contrast microscopy: ESCA (**a**), ESCA-2 (**b**), ESCA-3 (**c**), ESCA-5 (**d**). “-” scar bar = 100 μm. Passage number: all ESCA cell lines in this experiment are 15 th generation

**Fig. 2:**
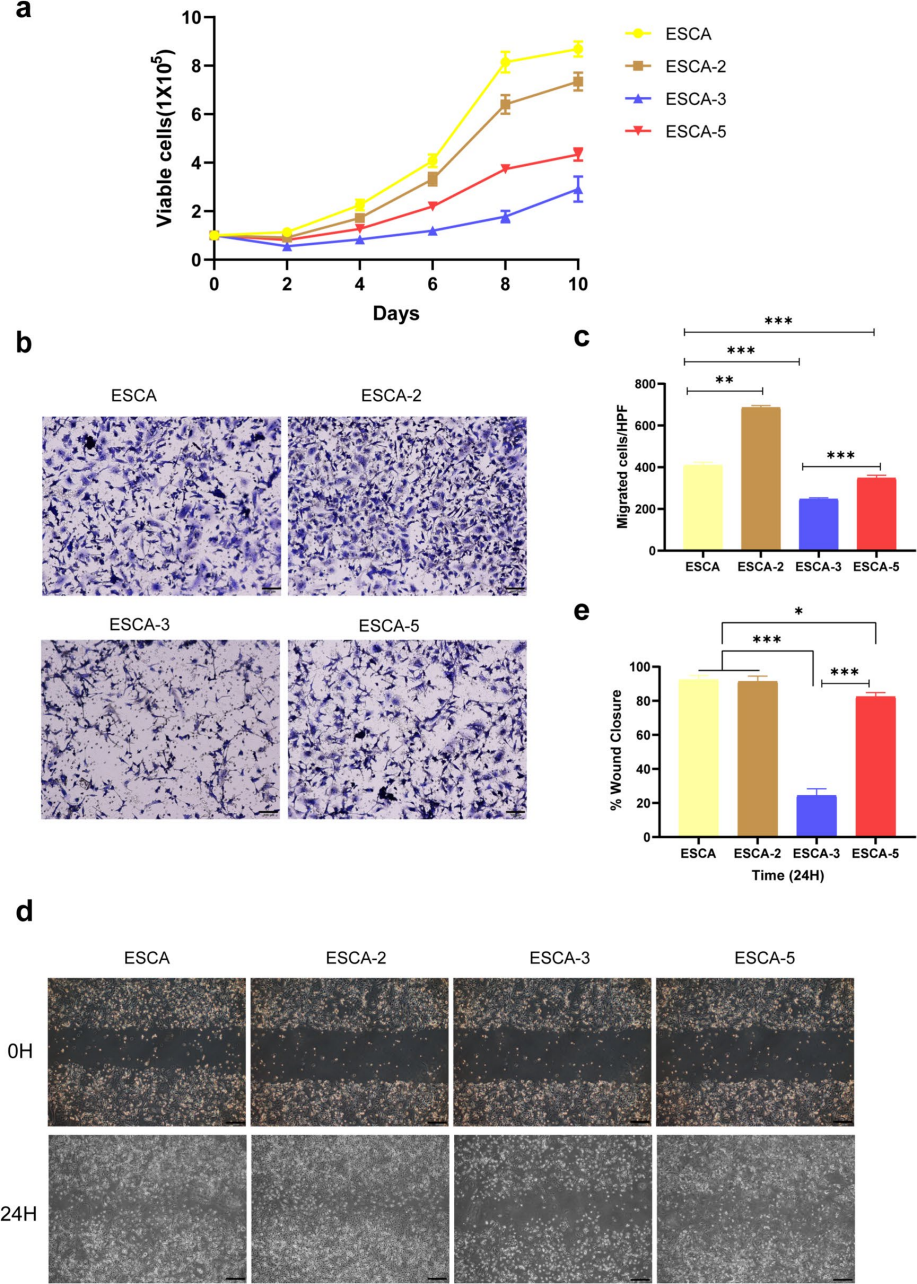
Growth characteristics and migration ability of ESCA and three sublines. Growth curves for different ESCA cell lines (**a**). Relative cell migratory ability of ESCA and its sublines cells was analyzed by Transwell assay (**b**), the quantitative analysis of the migrated cells (**c**), and wound healing experiments (**d**), the quantitative analysis of the wound closure were displayed (**e**). “-” scar bar = 100 μm. * *P* < 0.05, ** *P* < 0.01, *** *P* < 0.001, determined by one-way ANOVA analysis. H means hours. Passage number: 18–25 th generations were used

ESCA was established by mixing pre-stabilized sublines of ESCA-2, ESCA-3 and ESCA-5. Until now, all ESCA cell lines have been passaged to more than 60 generations. Figure 1 shows the distinct cell morphologies of ESCA and its three sublines. ESCA, ESCA-2 and ESCA-5 were characterized by polygonal spindle or irregular with much vacuolation in cytoplasm, while ESCA-3 was small round or oval cells with transparent cytoplasm (Fig. 1c). In terms of proliferation, ESCA had the fastest proliferation rate, followed by ESCA-2, ESCA-5, and ESCA-3 (Fig. 2a). The population doubling time for ESCA was 48–56 h, consistent with published data (Table 1). The cell migration ability of ESCA-3 cells was the weakest among ESCA and other types (Fig. 2b–e).

**Table 1:** Overview of established uterine carcinosarcoma cell lines

Cell line	Country	Age	Histology	DT(H)	CN	Transplant	Gene characteristics
FU-MMT-1 (1992) [15]	Japanese	75	AC, Rhabdomyosarcoma	26	47	Yes	N. R
FU-MMT-2 (1992) [15]	Japanese	61	Biphasic	30	80	No	N. R
EMTOKA (1993) [16]	Japanese	64	Biphasic	50	61–89	Yes	TP53
FU-MMT-3 (1997) [17]	Japanese	62	Rhabdomyosarcoma	29	74–80	Yes	c-MYC (+)
MT-213-VGH (2001) [18]	Chinese	62	N. R	24.29 ± 1.81	30–50	N. R	N. R
HTMMT (2004) [19]	Japanese	66	AC, Leiomyosarcoma,	60–72	87–100	Yes	PTEN, TP53
JHUCS-1 (2004) [20]	Japanese	57	Carcinomatous	30	43–47	Yes	N. R
CS-99 (2008) [21]	Germany	79	Sarcomatous	23–27	49–53	N. R	N. R
TU-ECS-1 (2017) [22]	Japanese	66	Endometrioid AC, Undifferentiated endometrial sarcoma	18.2 ± 2.1	44–49	Yes	KRAS TP53
ESCA (2024)	Chinese	58	Biphasic	48–56	52–58	Yes	TP53, TRRAP, ARHGAP35

*DT* Double time, *H* Hours, *CN* Chromosome number, *AC* Adenocarcinoma, *N. R* Not reported

### ESCA has severe chromosome karyotypic abnormalities

**Fig. 3:**
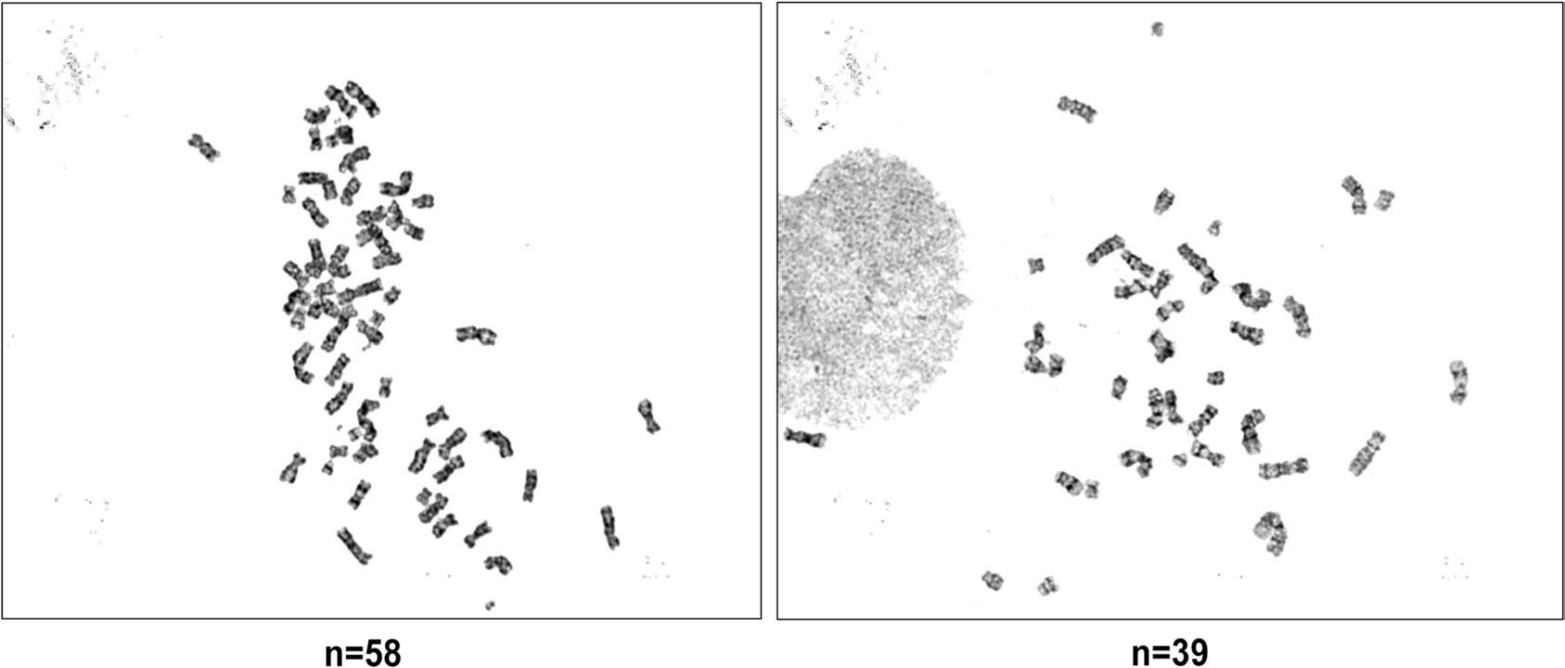
Karyotype of ESCA cells. Passage number: 20 th

A total of 10 metaphase cells of ESCA were examined for chromosome analysis. 20 split phases were observed under the microscope, and 5 were karyotyped. ESCA showed severe chromosome karyotype abnormalities, including ectopic, incorrect splicing, and abnormal number of chromosomes. The chromosome karyotypes of all split phases were inconsistent, 80% of them were 52–58 chromosomes, individual cells were less than 42 (20%), and the number of chromosomes exceeded the scope of G-band chart of karyotype analysis software (Fig. 3). It was confirmed that ESCA cells and its sublines were consistent and do not correspond to any cell lines in the public cell bank using STR analysis (Table 2).

**Table 2:** Results of STR analysis

Microsatellite (chromosome)	ESCA	ESCA-2	ESCA-3	ESCA-5
D5S818	11,12	11,12	11,12	11,12
D13S317	11,11	11,11	11,11	11,11
D7S820	11,11	11,11	11,11	11,11
D16S539	12,12	12,12	12,12	12,12
VWA	17,17	17,17	17,17	17,17
TH01	9,9	9,9	9,9	9,9
AMEL	X, X	X, X	X, X	X, X
TPOX	9,9	9,9	9,9	9,9
CSF1PO	10,12	10,12	10,12	10,12
D12S391	18,19	18,19	18,19	18,19
FGA	22,22	22,22	22,22	22,22
D2S1338	20,20	20,20	20,20	20,20
D21S11	30,31	30,31	30,31	30,31
D18S51	15,15	15,15	15,15	15,15
D8S1179	10,10	10,10	10,10	10,10
D3S1358	16,17	16,17	16,17	16,17
D6S1043	13,18	13,18	13,18	13,18
PENTAE	11,15	11,15	11,15	11,15
D19S433	13,13	13,13	13,13	13,13
PENTAD	9,10	9,10	9,10	9,10
D1S1656	16,18	16,18	16,18	16,18

### Immunofluorescence and immunocytochemistry

**Fig. 4:**
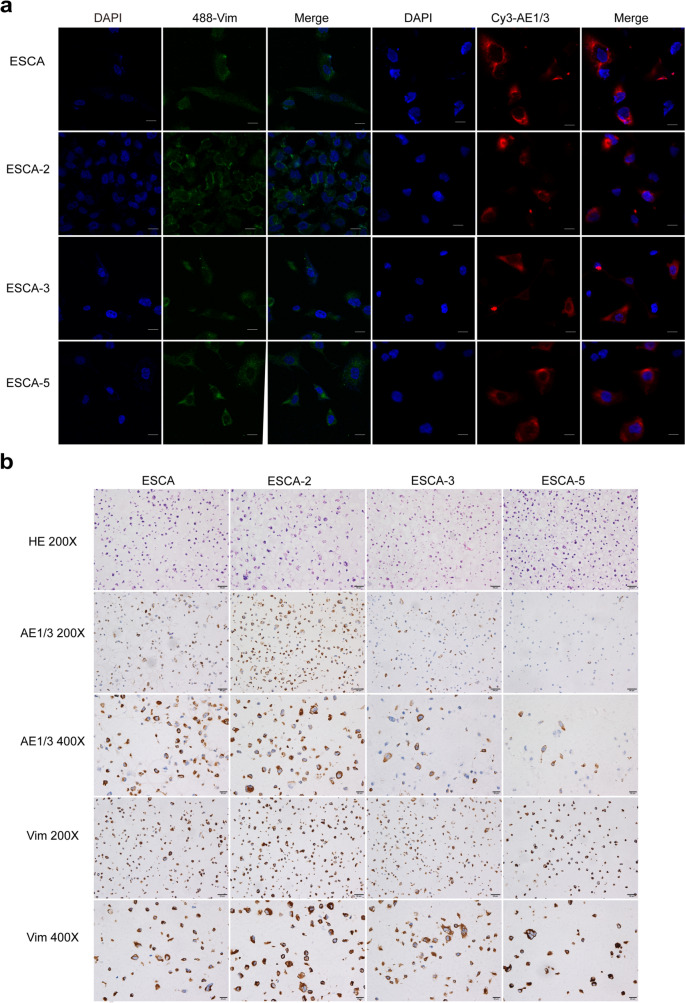
Immunofluorescent (**a**) and immunohistochemical (**b**) profiles of cytokeratin AE1/3 and Vimentin in cultured cells. “-” scar bar in white = 20 μm. Passage number: 29 th generation for IHC, and 32–36 th generation for immunofluorescent

Immunofluorescence staining showed that ESCA and its subline cells were positive for both epithelial marker cytokeratin AE1/3 and mesenchymal marker vimentin (Fig. 4a), which were consistent with the results of immunocytochemistry (Fig. 4b).

### ESCA sublines have different sensitivity to chemotherapeutic drugs

**Fig. 5:**
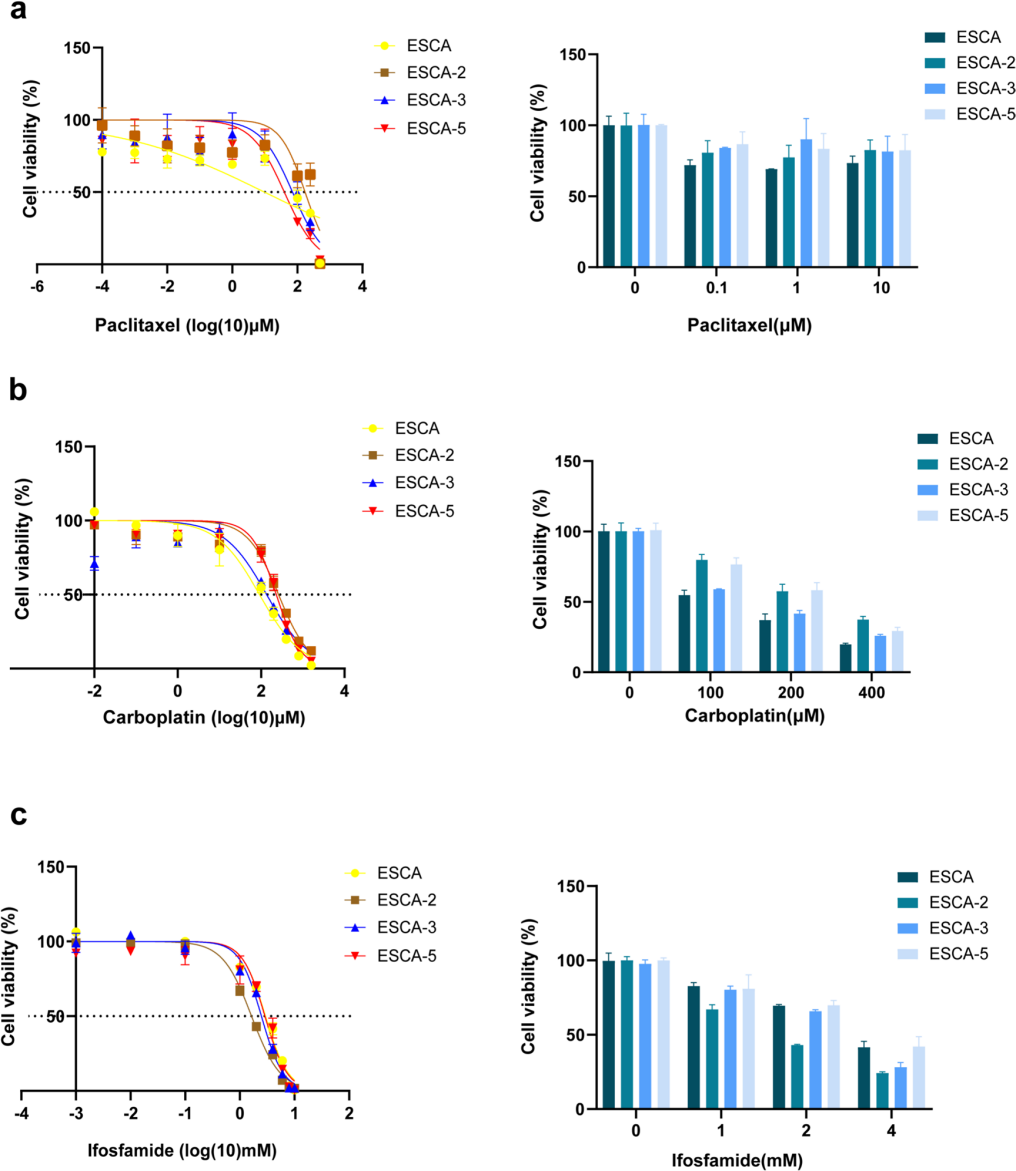
Chemosensitivities of the ESCA and its sublines cells to paclitaxel (**a**), carboplatin (**b**) and ifosfamide (**c**), the quantitative analysis of the cell viability was showed on the right. Passage number: 25–30 th generation of ESCA and its sublines were used in this experiment

The sensitivity of ESCA and its three sublines to paclitaxel, carboplatin and ifosfamide were detected. The results showed that ESCA-2 had the highest value of half maximal inhibitory concentration (IC-50) to paclitaxel (172.3 μM), followed by ESCA-3 (71.82 μM), ESCA-5 (39.79 μM), and ESCA (9.75 μM), suggesting that ESCA was most sensitive to paclitaxel (Fig. 5a). For carboplatin, ESCA (91.63 μM) had the highest sensitivity compared to other sublines (Fig. 5b). Conversely, ESCA-2 (1.640 mM) was most sensitive to ifosfamide than ESCA (2.944 mM), ESCA-3 (2.482 mM) and ESCA-5 (2.876 mM) (Fig. 5c).

### Comparison of molecular characteristics between different ESCA sublines

**Fig. 6:**
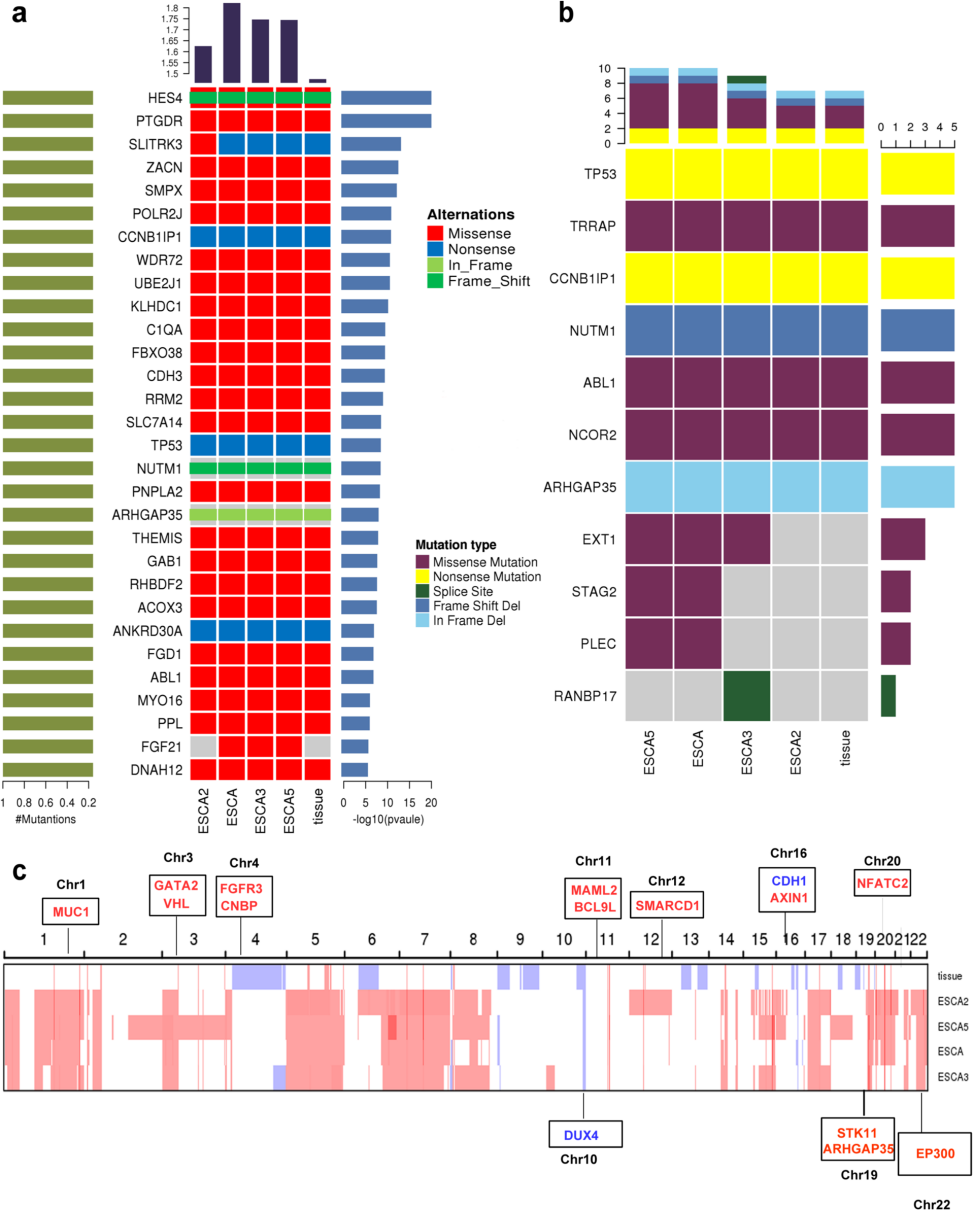
Results of whole-exome sequencing performed on patient’s tissue, ESCA and three sublines. Heat map of significantly mutated genes, which was significantly higher than the background mutation frequency, was shown in (**a**). The top bar chart shows each sample mutation burden (mutation rate). The bar chart (green) on the left side of the heat map shows the proportion of samples with this mutation in the total number of samples. The bar chart (blue) on the right side of the heat map shows the transformation of −log10(*P* value). The larger the value, the more significant the difference is. Heat map of driver genes variant was shown in (**b**). Copy number variant (CNV) analysis of the patient’s tissue, ESCA and its sublines was shown (**c**). The genes in red mean copy number gain, the genes in blue mean copy number loss. Passage number: ESCA, 28 th; ESCA-2, 30 th; ESCA-3, 26 th; ESCA-5, 26 th

Whole-exome sequencing (WES) was conducted on ESCA and its sublines, as well as the patient’s tissue block. Figure 6a shows significantly mutated genes of ESCA sublines and primary tumor based on somatic single nucleotide variants (SNVs) and insertions/deletions (InDels) in the somatic cells. It was observed that ESCA and its sublines shared certain somatic variations but mutational signature abundance was heterogenous. Specifically, the abundance of ESCA mutations was the highest, whereas ESCA-2 was the lowest. From the SNVs related to cancer, it was shown that ESCA sublines and patient’s primary tumor share some driven genes, including *TP53* (NM_001126115:exon1:c.G42 A:p.W14X),*TRRAP*(NM_003496:exon59:c.C9032 T:p.A3011 V),*CCNB1IP1*(NM_182849:exon5:c.C550 T:p.R184X),*NUTM1*(NM_001284293:exon7:c.2342_2352 del:p.G781fs)and*ARHGAP35*(NM_004491:exon1:c.1468_1470 del:p.490_490 del) (Fig. 6b). Interestingly, there had some difference in SNVs mutation between ESCA sublines. *EXT1*(NM_000127:exon1:c.A289G:p.K97E) mutation was not detected in ESCA-2. In addition, we detected some genetic variations in ESCA and ESCA-5 but not in ESCA-2 and ESCA-3 such as *STAG* ((NM_006603: exon29:c.G3152 T:p.G1051 V) and *PLEC* (NM_201378:exon31:c.C4615 T:p.R1539 W). Besides, *RANBP17(*NM_022897:exon22:c.2340-1G > T) mutation was only observed in ESCA-3 subline.

Some copy number variations (CNVs) were observed in the tissue block and all ESCA cell lines from Fig. 6c. Specifically, we noted an increase in somatic copy number, including *FGFR3* (4p16.3), *BCL9L* (11q23.3), *MUC1* (1q22), *GATA2* (3q21.3), *VHL* (3p25.3), and *SMARCD1* (12q13.12). While CNVs were decreased in *CDH1* (16q22.1) and *DUX4* (10q26.2).

### ESCA sublines have tumorigenicity and show different levels of glucose metabolism

**Fig. 7:**
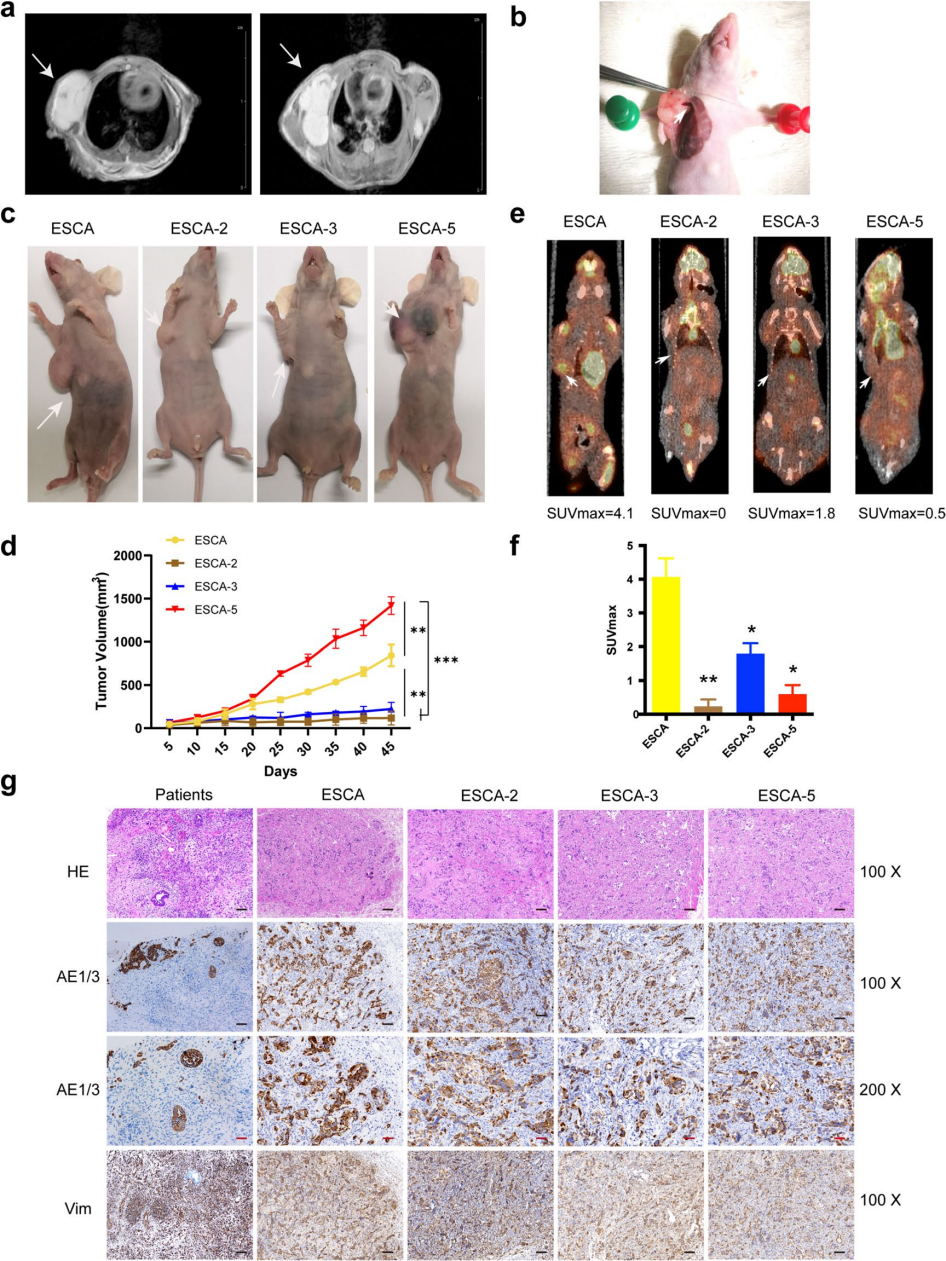
Tumorigenicity in the BALB/c-nu nude mice. Magnetic resonance imaging (MRI) of transplanted tumors was performed 22 days (**a**). The transplanted tumor was rich in blood supply, an invisible thick vessel penetrated deep into tumor. The vessel is indicated with arrows (**b**). ESCA and its sublines rapidly formed xenografts after inoculation into the BALB/c-nu nude mice (**c**). Growth curve of the transplanted tumors (**d**). Representative ^18^F-FDG micro-PET/CT imaging of tumor-bearing mice (**e**). The tumor SUVmax in xenograft mice with ESCA and its sublines (**f**). Histology and immunohistochemistry of ESCA cell lines, donor tumor and transplanted tumor (**g**). “-” scar bar in black = 100 μm, “-” scar bar in red = 50 μm. *P < 0.05, ** *P* < 0.01, *** *P* < 0.001, determined by One-way ANOVA analysis. Passage number: ESCA, 35 th; ESCA-2, 30 th; ESCA-3, 35 th; ESCA-5, 32 th

ESCA and three subline cells were subcutaneously injected into nude mice to assess tumorigenicity. Visible masses formed in all mice seven days post-injection. Figure 7a shows the magnetic resonance imaging (MRI) of transplanted tumors of two mice 22 days after injection. The infiltration of the right pleura highlighted the aggressive invasive potential of transplanted ESCA cells. Moreover, the tumor xenograft was rich in blood supply, an invisible thick vessel penetrated deep into tumor is displayed in Fig. 7b. Consistently, we found that ESCA-2, 3 and 5 were capable of tumor initiation in *vivo*.

Interestingly, contrary to cell proliferation rate, the transplanted tumor from ESCA-5 exhibited the fastest proliferation, followed by ESCA, while those from ESCA-2 and ESCA-3 showed minimal differences in transplanted tumor growth, as depicted in Fig. 7c, d. Furthermore, the micro-PET/CT scanning was carried out to evaluate the glucose metabolism of transplanted tumors 25 days post-injection. However, the transplanted tumor of ESCA-5 displayed highest proliferation rate although, the glucose uptake level was even lower than that from ESCA-3, and the transplanted tumor of ESCA presented the strongest glucose uptake (Fig. 7e, f). In addition, the xenografts morphologically mimicked the original tumor in HE staining. Both the transplant and primary tumors showed local intensive positivity for AE1/3, and diffuse intensive positivity for Vimentin (Fig. 7g).

## Discussion

Patients with UCS typically experience a poor clinical course due to its highly aggressive nature and high recurrence rate [23]. Most studies on UCS have been conducted using clinical samples, aiming to investigate its histological origin or molecular characteristics, to understand the reasons for its poor prognosis and identify strategies to improve clinical outcomes. Horizontal and microscopical studies were included [10, 24]. Nevertheless, some basic researches have explored the epithelial–mesenchymal transition of UCS using endometrial carcinoma cells as a tool [25, 26]. The study progression of UCS was limited not only due to the complexity of tumor itself, but to the lack of mature cell lines. Here, we have established a novel UCS cell line named ESCA, which can be indefinitely propagated in culture. Importantly, ESCA and its three sublines demonstrated tumorigenicity in vivo, with the transplanted tumors displaying high aggressiveness. And ESCA cell line exhibits histopathological characteristics consistent with the original tumor and appear to retain the nature of the original tumor. In addition, we isolated and established three subline cell lines from ESCA by collecting the precipitation at different time in primary culture, which retains molecular signature and conserves heterogeneity of UCS original tissue to the greatest extent. Thus, ESCA and its sublines may represent scientific tools for basic and pre-clinical researches of UCS.

Chemotherapy is an indispensable component of systemic therapy in many cases of UCS based on current guidelines. Studies of monotherapy regime for UCS patients reported that the response rates of platinum-based, paclitaxel, and ifosfamide were 18, 18, and 29%, respectively[27–29]. Although chemotherapy regimens (paclitaxel-carboplatin or paclitaxel-ifosfamide) are still debate in the treatment of advanced staged and recurrent UCS, the therapeutic efficacy of combined agents (50–60%) is superior to that of single agent chemotherapy. But the drug resistance still remains a serious problem for patients with UCS [23]. Chemosensitivity assays in our analysis revealed that differential sensitivities of ESCA and its three sublines to the anticancer agent paclitaxel, carboplatin, and ifosfamide. These results, on the one hand, confirmed that UCS is a highly heterogeneous disease with different sensitivity to chemotherapy even cells from the same woman and combination therapies are necessary. Besides, established ESCA and its sublines preserved the nature of chemoresistance and would be valuable tool for elucidate the chemoresistance mechanisms in the future.

Despite the improved knowledge acquired in recent years, the main processes underlying UCS pathogenesis remain controversial. “Collision” and “combination” theories were major hypotheses in the past [30, 31]. Currently, most scholars believe that UCS is monoclonal derived from a single cell of origin, which comes from EC, and then develops the characteristics of sarcoma through trans-differentiation, which is known as conversion model [12]. This theory, to a certain extent, might also explain the characteristic clinical behavior and therapeutic response of this neoplasm, which resembles high-grade EC rather than other sarcomas [32]. Travaglino et al. analyzed the genomic profile of UCS using The Cancer Genome Atlas (TCGA)-2013 classification for endometrial adenocarcinoma. They found that the majority of UCS tumors (73.9%) were in the high copy-number/TP53 mutated category with poor prognosis[33]. This finding aligns closely with the results obtained in our present study. Through the analysis of the molecular characteristics of ESCA cell lines and their original tumor, it is clarified that all ESCA cell lines have oncogenic *TP53* mutation but not *PIK3 CA* mutation. Besides, transformation/transcription domain-associated protein (TRRAP), a constituent of several histone acetyltransferase (HAT) complexes, is a critical positive regulator of both mutant type p53 and wild-type p53 levels in cancer entities. In addition, through its association with various HAT complexes, *TRRAP* is involved in most DNA transaction process, including transcription, replication, and repair. Therefore, we can speculate that the missense mutation of *TRRAP* play a critical role in development of UCS, but requires further validation. In the aspect of CNV, ESCA sublines have the amplification of *FGFR3* and *BCL9L* genes, which is roughly consistent with the study Levein et al. [10]. In conclusion, the molecular abnormalities of ESCA are mainly reflected in the high level of stemness and epithelial mesenchymal transition and DNA damage and repair.

By comparing three sublines of ESCA, this study attempts to raise the following points. Although the proliferation rate of ESCA-5 is low, the growth rate of transplanted tumor surpasses that of all other sublines. The proliferation rate of ESCA transplanted tumor is higher than that of ESCA-2 and ESCA-3, suggesting that heterogeneous tumor cells of different types of tumor cells promote each other. Most malignant tumors depend on anaerobic glycolysis to grow rapidly, so ^18^F-FDG exploits this [34]. The level of SUV uptake is seriously inconsistent with the tumor proliferation rate, suggesting that there are perhaps more important energy supplies than glucose in the malignant process of carcinosarcoma, which need to further investigation. It also suggested that SUVmax may not be a useful biomarker for reflecting UCS aggressiveness. Previously studies had demonstrated that higher SUVmax of primary EC showed significantly poorer prognosis than those with a low SUVmax [35, 36], but for patients with UCS, SUVmax was not associated with survival rate and clinicopathological prognostic factors [37, 38]. Thus, using only ^18^F-FDG PET/CT may not be the best way to assess the extent of UCS tumor infiltration. This current study had certain limitations. The isolation of ESCA three sublines was a physical method, which on the basis of cells’ different sedimentation rate in primary culture, whether the isolation method could be applied to the other malignancies still need to be further explored and verified. Besides, there are currently scare commercial uterine carcinosarcoma cell lines, and has no control mature cell lines to compare with ESCA and its sublines concerning growth characteristic and chemotherapy sensitivity.

In a word, a newly uterine carcinosarcoma cell line named ESCA and its three sublines were established from a Mainland Chinese patient. All ESCA sublines showed fast and unlimited multiplication until now and tumorigenicity in vivo. Besides, WES showed that it carries gene mutations of *TP53*, *TRRAP* mutations and amplification of *FGFR3* and *BCL9L* genes. This cell line, therefore, may be an ideal tool to analyze the biological and molecular characteristics of UCS, as well as facilitate the establishment of treatment strategies to improve the prognosis of advanced UCS patients.

## Data Availability

All data that support the findings of this study are available from the corresponding author upon request.
